# Air Pollution and Migration Decision of Migrants in Low-Carbon Society

**DOI:** 10.3390/ijerph20010870

**Published:** 2023-01-03

**Authors:** Feiwei Shen, Qiang Wang, Jing Zou, Huili Yan, Baitao Wang

**Affiliations:** 1 College of Public Administration, Hangzhou Normal University, Hangzhou 311121, China; 2 Hangzhou International Urbanology Research Center and Zhejiang Urban Governance Studies Center, Hangzhou 311121, China; 3 School of Urban and Regional Science, East China Normal University, Dongchuan Road 500, Shanghai 200241, China; 4 The Centre for Modern Chinese City Studies, East China Normal University, North Zhongshan Road 3663, Shanghai 200062, China; 5 School of Finance, Zhejiang University of Finance and Economics, Hangzhou 310018, China; 6 School of Tourism, Hainan University, Haikou 570228, China

**Keywords:** air pollution, migrants, migration decisions, conditional logit model, IV estimation

## Abstract

The influence of environmental quality on the quality of urban life and on migration decisions is an important research issue in urban economics and environmental economics. Using the 2012–2014 China Labor Dynamics Survey data (CLDS), this paper uses a conditional logit model (CLM) and Instrumental Variable (IV) estimation to examine the impact of air pollution on the migrant migration decision. We find that air pollution significantly negatively impacts the migration decisions of migrants. Specifically, if the PM2.5 level of a city increases by 10 μg/m^3^, the probability of migrants flowing into the city will be significantly reduced by 21.2%. It shows that migrants choose to flow into cities with better spatial quality to reduce the risk of exposure to air pollution. After controlling for the characteristics of the outflow and the reasons for the flow, the impact of air pollution on migrants’ migration decisions remains robust. Heterogeneity analysis shows that middle-aged, male, married, and highly educated migrants are more sensitive to air pollution. This paper enriches the research on air pollution and labor mobility at the micro level and provides empirical evidence for policymaking related to environmental governance and labor mobility in a low-carbon society.

## 1. Introduction

In modern society, more and more residents advocate the low-carbon life. China’s early extensive economic development model has caused a concentrated outbreak of environmental hazards in recent years. The “2017 China Environmental Bulletin” shows that 239 of the 338 prefecture-level and above cities across the country have exceeded environmental air quality standards, and the proportion of cities exceeds 70.7%. Severe air pollution has not only reduced the Chinese urban amenity but also put more significant pressure on the health of residents. Air pollution differs from favorable urban amenities such as a comfortable climate and ample green areas, and it is an urban disamenity. Studies have shown that air pollution not only directly affects the physical health of residents, such as causes heart disease, respiratory diseases, and shortened life expectancy [1,2,3], but also has a negative impact on residents’ mental health, such as reducing residents’ subjective well-being and mental health [4,5]. Out of consideration for the quality of life and physical health, more and more residents choose to move out of cities with severe air pollution. According to Rosen-Roback’s spatial equilibrium theory, the spatial mobility of migrants is affected by income, cost of living, and urban amenities [6]. However, as an essential factor of urban amenities, air quality has often been overlooked in previous studies. With people’s pursuit of good environmental quality, the introduction of environmental indicators has important theoretical significance for accurately understanding migrants’ migration decisions.

Based on the above theoretical and practical background, this paper first uses global PM2.5 satellite raster data to calculate PM2.5 concentration data in 267 cities at the prefecture level and above in China from 2012 to 2014 as a measure of urban air pollution; secondly, using the data from the 2012–2014 China Labor Force Dynamics Survey (CLDS), this research has constructed a dataset of migrants’ migration decisions, with 267 cities at prefecture-level and above as destination selection sets. On this basis, this paper investigates the effect of air pollution on the probability of migrants’ migration decisions using the conditional logit model. This paper uses the ventilation coefficient as an instrumental variable of air pollution to address the endogeneity problem, which is widely used in related fields [7,8].

The research contributions of this article are mainly reflected in the following points: First, although the migrants’ migration decisions have been extensively studied [9,10,11], as far as we know, few empirical analyses show that air pollution is a critical factor in the migrants’ migration decisions. In order to narrow this gap, we expanded this research question. Based on Rosen-Roebuck’s spatial equilibrium theory, this paper explores the relationship between air pollution and migrants’ migration decisions while controlling socioeconomic, demographic, and unobserved heterogeneity. Second, once a migrant decides to leave the place of residence, he/she faces a series of alternative cities when deciding on the destination city. Unlike other articles, this paper uses the conditional logit model to test the relationship between air pollution and the migration decision of the floating population. Considering that air pollution is endogenous and omitted variables may bias estimates, this paper uses the ventilation coefficient as an instrument for air pollution. Further, the conditional logit model is nonlinear, and Two Stage Least Square (2SLS) is no longer appropriate, so the control function method is applied to deal with the problem. Third, most existing studies on air pollution and the flow of migrants use aggregated data or statistical data for empirical analysis [12,13]. However, these data ignore the differences in individual characteristics and cannot explore the heterogeneous impact of air pollution on migrants’ migration decisions. Existing studies have found that the heterogeneity of environmental effects is significant for understanding the individual’s response to environmental effects [14]. Differences in responses to similar changes in environmental quality may mean systematic differences in individual adaptation strategies to deal with air pollution. Studying the response patterns between different individual groups may help formulate effective policies to reduce the negative effects of environmental pollution. This paper uses global PM2.5 satellite raster data and Chinese labor force dynamic survey data (CLDS) to accurately identify the impact of air pollution on migrants’ migration decisions and analyze its heterogeneity. The heterogeneity research conclusions of this article provide an empirical basis for local governments to make policies to attract migrants by improving environmental quality.

The rest of this paper is arranged as follows. Section 2 describes the literature review, Section 3 shows the methodology and data, the empirical results are shown in Section 4, including the baseline regression results, endogenous solving, robustness check, and heterogeneity analysis, and the last two sections contains the discussion and conclusion.

## 2. Literature Review

There are two main types of literature on the impact of air pollution on migration behavior. The first type of literature relates to Tiebout’s “voting with their feet” theory. As early as 1956, Tiebout believed that people would adopt the “voting with their feet” method and flow to places where the public policies best matched their personal preferences, forcing local governments to compete and improve social governance [15]. On this basis, urban economists, based on Tiebout’s “voting with their feet” theory, have shown that urban public services such as educational resources, infrastructure, and health care have a significant impact on migrants’ migration decisions [16,17,18].

As an essential component of urban amenities, the impact of air quality on migrants’ migration has also gradually attracted the attention of scholars. Cameron and McConnaha (2006) conducted a microscopic study of the American census tract data and found that residents are sensitive to the environment [19]. Banzhaf and Walsh (2008) found that people “vote with their feet” on environmental quality, and there are both scale and structure effects [20]. Chen et al. (2022) investigated the impact of air pollution on population migration from the perspective of population emigration places based on the sample data of China’s population census and found that air pollution promotes population outflow by calculating the population emigration rate at the county level [21]. Li et al. (2017) found that air pollution significantly impacts labor migration, and educated male groups are more sensitive to air pollution [22]. Cui et al. (2019) used smartphone positioning data to study how air pollution dose affects urban population outflow, and the degree of impact is heterogeneous in different festivals [23]. Qin and Zhu (2018) analyzed the Baidu search index of the keyword “immigration” and found that when the air quality index increased by 100, the frequency of people searching for “immigration” would increase by 2.3%–4.8% the next day [24]. This result reflects the impact of air pollution on people’s willingness to emigrate. Based on smart meters’ big family data and using the fixed effect panel model, Wang (2021) found that the effect of air pollution on population migration is a short-term cumulative effect and that past experiences of air pollution continue to influence residents’ current migration behavior. The above studies directly investigate the impact of air pollution on migrants. However, some studies have also found that environmental pollution can negatively impact housing prices and the attractiveness of cities to labor [25,26].

The second type of research related to this paper discusses the negative effect of environmental pollution. Some scholars study the harm of air pollution to individuals from a microscopic perspective. When air pollution is severe, the crime rate of individuals affected by air pollution will increase, which shows significant heterogeneity at different temperatures [27]. Using the geolocation of crimes and wind direction as a source of pollution variation, air pollution increases violent crime in both Chicago and Los Angeles [28]. Failure to adjust for the health of those who die will overstate mortality reduction benefits of decreases in air pollution [29]. In addition, the damage of air pollution to human health has also been a broad concern in the academic world [30,31]. The long-term exposure to air pollution has a significant effect on mortality by leveraging quasi-random variation in pollution levels generated by wind patterns near major highways [32]. Air pollution can increase mortality rates [33]. In addition, air pollution has a negative impact on residents’ mental health. Air pollution not only significantly reduces residents’ subjective well-being [5,22], but also has a negative effect on residents’ mental health [34,35]. Air pollution directly harms the human health of residents and may lead to respiratory infection, cerebrovascular disease, lung infection, and other diseases [3,13,36]. Long-term exposure of pregnant women to air pollution increases risks, such as early pregnancy abortion [37], low birth weight [38], and premature delivery [39]. Severe air pollution can also affect sleep quality [40] and even lead to the premature death of people after a long time of influence [41,42].

As air pollution seriously harms residents’ physical health and psychological activities, people change their living habits to cope with air pollution, including adaptive protection and active escape [43]. People will have short-term protective behaviors on days with severe air pollution to reduce pollution harm to physical and mental health [44], such as reducing outdoor activities [45], shortening labor supply [46,47], purchasing anti-haze masks and purifiers, and a series of other methods [48]. Active flight behavior refers to residents leaving their residential areas due to air pollution, such as emigration and moving out of the city [49]. Under the condition that other external environments remain unchanged when the damage caused by air pollution to human health becomes increasingly high or even exceeds the utility obtained in the environment, residents may migrate to create a better personal environment for themselves [21].

In summary, there are still few studies on the impact of air pollution on migrants, especially on migrants’ migration decisions. Existing studies on the impact of air pollution on migrants’ migration decisions are mainly based on local or city-level statistical data, and empirical analysis is carried out by designing a panel data model. Since individual characteristics of migrants cannot be directly observed or controlled, an implicit assumption in the setting of such models is that the location preference of migrants does not change with time and space, so that the impact of air pollution and other location characteristics on migrants’ migration decision can be obtained. However, if the demographic characteristics of the migrants change at some point in time, resulting in an increase or decrease in the average preference of the migrants for air pollution, the adoption of the panel data model will face the problem of missing variables. Given the limitations of existing research, this paper establishes a conditional logit model at the micro-individual level. It uses the instrumental variable method to solve the estimation bias caused by the missing variables at the city level, which can more accurately measure the effect of air pollution on the migrants’ migration decisions in China.

## 3. Methodology and Data

### 3.1. Methodology

#### 3.1.1. Conditional Logit Model

In this paper, once a migrant decides to leave the place of residence, he/she faces a series of alternative cities when deciding on the destination city. That is to say, the choices faced by the floating population are diverse. A multinomial or a conditional logit model can be used for estimation involving multiple selections. The multinomial logit model can only consider independent variables that do not vary over alternatives, while the conditional logit model can examine the effect of alternative-varying variables [50]. The core explanatory variable of this paper, air pollution, is an alternative-varying variable. Therefore, this paper uses the conditional logit model to test the relationship between air pollution and the migration decision of the floating population. Furthermore, the conditional logit model, developed based on the random utility model, is particularly appropriate when microscopic survey data are in use. The model has a solid microeconomic foundation and enables us to identify how an individual makes locational decision in a utility maximization framework.

This study examines the migrants’ migration decisions within a utility maximizing, discrete choice model that incorporates city attributes variables and a vector of control variables. As in the conditional logit model, the independent variable only contains the attributes related to the scheme attributes and does not contain any information related to the decision-making subject. Therefore, individual characteristics are not shown in the equation, as the dimension of the later heterogeneity analysis. The utility an individual i derives by choosing destination j takes the form:

(1) $$U_{ij}=\alpha {PM}_{2.5ij}+\beta Z_{ij}+\varepsilon_{ij}(i=1,2,\ldots \ldots ,N;j=1,2,\ldots \ldots ,J)$$

where PM_2.5ij_ represents the concentration of air pollution of the alternative city j facing by individual i, Z_ij_ is the vectors of other characteristics of alternative city j facing by individual i, and ε_ij_ represents a set of random errors. Faced with J alternative cities, individual i will choose destination j on the condition that the utility of destination j (U_ij_) exceeds that of any other destination (U_ik_):

*P_ij_* = *P_r_*[*U_ij_* > *U_ik_*] ∀*k*, where *k* ≠ *j* (2)

According to McFadden (1974) [51], if the ε_ij_ follows the assumption independence of irrelevant alternatives (IIA), then the probability that city *j* is chosen by individual *i* is the conditional logit:

(3) $$Prob\;\left(choice_{ij}=1|PM_{2.5},X\right)=\frac{\exp\left(\alpha_{1}PM_{2.5}{}_{ij}+\beta Z_{ij}+\varepsilon_{ij}\right)}{\sum_{j=1}^{J}\exp\left(\alpha_{1}PM_{2.5}{}_{ij}+\beta Z_{ij}+\varepsilon_{ij}\right)}$$

where *choice_ij_* is a dummy variable; if individual *i* selects city *j*, *choice_ij_* is 1. Otherwise, *choice_ij_* is 0. Note that the conditional logit model is a discrete choice model in which the independent variables can only be alternative-specific variables and cannot be added directly to individual characteristic variables. Therefore, to study individual heterogeneity, this paper will divide the samples into different groups by individual characteristics. As mentioned before, the testable hypothesis of this paper is that the higher the concentration of air pollution in city *j*, the lower the probability that individual *i* chooses to move to city *j*, that is, *α*_1_ < 0.

#### 3.1.2. Instrumental Variable Design

Although the conditional logit model can control individual fixed effects and avoid the lack of individual-level variables, the factors that affect migrants’ migration decisions at the urban level are complex, and there may be other unobservable factors. These factors will simultaneously affect the air pollution of the candidate cities and the migration of migrants; that is, there is a problem of omitted variables. In addition, since air pollution is primarily affected by urban economic activities and population aggregation, control variables such as urban economy, population size, and public services will strongly correlate with local air pollution levels. Therefore, we adopt an instrumental variable (IV) approach to address the endogeneity issue such as omitted variables and reverse causality. 

We utilize ventilation coefficient (*VC_it_*) to construct the instrumental variable [8,21]. First, the ventilation coefficient can directly affect the diffusion and dispersion of pollutants in the lower atmosphere, satisfying the assumption of instrumental variables. Secondly, the ventilation coefficient is affected by the wind speed and atmospheric boundary layer height and is not affected by human economic activities, which satisfies the exogenous assumption of instrumental variables. Drawing from the method adopted by Hering et al. (2014) [8], we construct IV as follows:

(4) $$VC_{it}=WS_{it}\times BLH_{it}$$

where *WS_it_* and *BLH_it_* represent wind speed and atmospheric boundary layer height, respectively. The original data of wind speed and atmospheric boundary layer height are obtained from the European Centre for Medium-Range Weather Forecasts (ECMWF).

#### 3.1.3. Control Function Method

Since the conditional logit model is a nonlinear model, estimation by 2SLS with IV is not appropriate. Therefore, this paper will run the regression by using the control function method in the nonlinear model with continuous endogenous explanatory variables [52]. The steps are as follows:

choice = 1[*β*_1_*X*_1_ + *α*_1_*PM*_2.5_ + *μ*_1_≥0] (5)

PM_2.5_ = *β*_2_*X*_2_ +*υ*_2_ (6)

where *X*_1_ is city-specific variables except for the concentration of air pollution, and *X*_2_ contains *X*_1_ and the instrument variable. To deal with the omitted variable bias, the estimation can be divided into two steps. 

Step1: Let μ_1_ be a control function of υ_2_. The simplest way is to specify the control function as linear in υ_2_. Since υ_2_ is unobservable, it is necessary to perform Ordinary Least Squares (OLS) regression on the instrumental variable VC_it_ to obtain the residuals ${\hat{v}}_{2}$. 

Step2: Put the estimated value of the residual term into the conditional logit model, that is, regressing choice_ij_ on the PM_2.5_, ${\hat{v}}_{2},$ and other control variables (see Equation (8)) to obtain the unbiased α1 estimate [10].

(7) $$\mu 1_{\;}=\mathrm{CF}(\upsilon_{2},\;\lambda )+\tilde{\mu_{1}}=\lambda \upsilon_{2}+\tilde{\mu_{1}}$$

(8) $$\mathrm{choice}=1[\beta_{1}X_{1}+\alpha_{1}{\mathrm{PM}}_{2.5}+\mathrm{CF}(\upsilon_{2},\;\lambda )+\tilde{\mu_{1}}\geq 0]$$

### 3.2. Data

#### 3.2.1. Migrants

The data of migrants are collected from the “China Labor Force Dynamic Survey (CLDS)” conducted by the Center for Social Survey at Sun Yat-sen University. This survey adopts a multi-stage stratified probability proportionate to size sampling (PPS) method to interview households from 29 provinces in China (Hong Kong, Macau, Taiwan, Tibet, and Hainan were excluded). It interviews all laborers between 15 and 64 and asks for their basic personal information, education experiences, and working conditions. CLDS defines the labor force that crosses the county level and above administrative units for more than six months as the floating population and records the year of their migration, the place of inflow, and the reason for their migration. This paper regards migrants’ last migration destination as their final choice. For example, if Beijing is the final city into which a migrant flows, then the place of household registration to Beijing is the final migration decision, i.e., choice_ij_ = 1. The other 266 cities have choice_ij_ =0, because the other 266 cities are candidates.

From the data processing, there are 2387 migration samples in 2012 and 4286 migration samples in 2014. After removing samples without migration destination information and matching them with municipal data, the number of migration samples is 6115, and the city sample is 267. Therefore, the total sample is 1,632,705. In addition, considering that the data only have two periods, the paper will not treat it as panel data. The descriptive statistics of core variables are as follows, including age, gender, marriage, education level, and self-rated family level, and other city characteristics (Table 1).

#### 3.2.2. City Characteristics

After matching the city-level data with labor migration data, they eventually cover 267 prefecture-level cities in China. This paper divides city-level variables into four categories. The first category is environmental variables, including the concentration of PM_2.5_, ventilation coefficient, average temperature, and precipitation. PM_2.5_ concentrations come from the annual world PM_2.5_ data from 1998 to 2016 released by Columbia University. This is the proxy variable of air pollution. The higher the PM_2.5_ concentration in a city, the more serious the air pollution. The instrumental variable is the ventilation coefficient, which comes from the European Centre for Medium-Range Weather Forecasts (ECMWF). The information is extracted by using the city’s latitude and longitude. Weather variables, including temperature and precipitation, also affect migration decision. At the same time, temperature and precipitation are closely related to air pollution. Therefore, they should be added to reduce the omitted variable problem. Besides, every city’s ventilation coefficient may be related to local climate conditions. If the paper does not control temperature and precipitation, then the influence of temperature and precipitation on labor migration might enter the ventilation coefficient. That is, the instrument may no longer be exogenous. The weather data are obtained from annual and daily meteorological datasets released by the China Meteorological Data Sharing Service System (CMDSSS).

The second category is economic variables, including the city’s average wages of employees and industrial structure. The average wage of employees in a city represents the expected wage level of workers who move into this city. It is the main driving force for population migration. The average wage of employees is deflated by the consumer price index, based on 1978, to eliminate the impact of inflation. The industrial structure is calculated by the proportion of the secondary and tertiary industries in Gross Domestic Product (GDP). From the literature review, cities with a high proportion of non-agricultural industries usually have an advanced economy. In addition, the non-agricultural industry can create more employment opportunities than the agricultural industry, so this paper predicts that the higher the proportion of the non-agricultural industry is, the more migrants the city attracts.

Based on Tiebout’s “voting by their feet” theory, migrants may choose the city that provides better public services. The third kind of variable is related to public services, including educational and medical levels. A city’s educational level is expressed by the number of teachers in primary and secondary schools divided by the city’s population, that is, the number of teachers per 1000 people. A city’s medical level is expressed by the number of hospital beds divided by the city’s population, that is, the number of beds per 1000 people. The city’s economy and public services data are obtained from the “China City Statistical Yearbook” and CEIC database.

Finally, this paper also adds the variable of household registered population, which does not include the migrant population. On the one hand, it is used to study the impact of population size on migrants’ migration. On the other hand, some cities, such as Haixi Mongolian and Tibetan Autonomous Prefecture, have high average wages and educational and medical levels. However, it is not because of the development of those cities. It is because their population is small that average values are high. Therefore, the estimates may be biased if the population is not controlled. Population data come from the “China City Statistical Yearbook” and the CEIC database. In addition, a dummy variable of whether the destination is a provincial capital city is added. Cities with higher administrative levels may have more policy advantages and development opportunities, thereby attracting more migration inflows. Note that all city-specific variables, except the dummy variables, are measured as averages from 2012 to 2014. Note that due to the dispersion of migration times and missing data for some years, all city variables, except dummy variables, are measured using the average values from 2012 to 2014.

## 4. Empirical Results

### 4.1. Baseline Regression Results

Table 2 reports the results of conditional logit regression excluding the instrumental variable. From Model (1) to Model (3), the effect of air pollution on migrants’ migration decisions is identified gradually by adding more control variables. Wald tests of the three regressions show that all variables are jointly significant at the 1% level. Meanwhile, for the samples used in this paper, the Chi^2^ of the Hausman test is very small or negative. Therefore, the null hypothesis cannot be rejected, and the difference in coefficients is not systematic. The assumption is not violated, and the results from conditional logit regression are credible. In addition, considering the problem of heteroscedasticity, the standard deviations of coefficients reported in this paper are all robust (White) standard errors.

Since the meaning of the raw coefficient is difficult to explain, this paper also reports the percent changes in odds for a unit increase in independent variables. Assuming that for migrant *i*, the probability of choice_j_ = l is *π_i_* and the probability of choice_i_ = 0 is (1 − *π_i_*), then the odds are the ratio of these two, which is shown in Formula (9). The closer the probability is *π_i_* to 1, the closer the odds are to +∞. In other words, the greater the odds for a city, the larger the probability that migrant i chooses to move to that city. The results can be interpreted as that holding all other variables constant, and the odds will change by a factor of exp (*β*_k_) for a unit change in *x_k_*. If exp (*β*_k_) > l, the odds will be “exp (*β*_k_) times larger”, and the probability of migrating to a certain city is also larger. Otherwise, if exp (*β*_k_) < l, the probability will become smaller. The relationship between the odds and raw coefficients can be expressed as Equations (10) and (11).

(9) $$odds=\Omega_{i}=\frac{\pi_{i}}{1-\pi_{i}}$$

(10) $$\mathrm{factor}\;\mathrm{change}\;\mathrm{in}\;\mathrm{odds}=\mathrm{odds}\;\mathrm{ratio}=\frac{\Omega \left(x_{1}x_{k}+1\right)}{\Omega \left(x_{1}x_{k}\right)}=\exp\;(\beta_{k})$$

(11) $$\%\;\mathrm{change}\;\mathrm{in}\;\mathrm{odds}\;=\left[\frac{\Omega \left(x_{1}x_{k}+1\right)}{\Omega \left(x_{1}x_{k}\right)}-1\right]\times 100\%=\left[\exp\left(\beta_{k}\right)-1\right]\times 100\%$$

In model (1), only environmental variables are added. The results show that the air pollution coefficient is positive, contrary to expectations. In model (2), economic variables are added, and the coefficient of air pollution tums negative. In model (3), all city-specific variables are added, and the coefficient of air pollution remains negative. By adding more control variables, the coefficient of air pollution changes from positive to negative, indicating that potential omitted variables tend to underestimate the adverse effects of air pollution. The direction of omitted variable bias is consistent with the previous analysis. The results from the model (3) show that by holding all other variables constant, increasing the PM2.5 concentration by 10 μg/m^3^ for a given city decreases the odds of choosing that city by 9.7%, significantly indicating that migrants prefer to choose the cities with better air quality to reduce the risk of air pollution exposure.

Temperature and precipitation also significantly affect the selection of migration destinations. In cities with a warm and humid climate, people may feel more comfortable and thus attract more migration inflows. The impact of economic and public service variables is also in line with expectations. A higher wage level or a more significant proportion of non-agricultural industries increases the probability that migrants choose this city, indicating that wage and employment opportunities are attractive to migrants. The coefficients of educational and medical levels are also significantly positive, indicating that people might “vote with their feet” on public services and choose to move to cities with better education and healthcare.

Besides, the population size and whether the destination city is the provincial capital positively correlate with the probability of migrants choosing to flow into this city. It implies that the migrant population is more likely to gather in large cities. The city with larger population sizes is more accessible to form a scale effect regarding public service supply, production, and consumption. Hence, this kind of city can attract more migration inflows. The city with a higher administrative level also has more policy privilege and then influences migrant’s choices.

China’s early and extensive economic growth model caused fast-growing cities to have more severe air pollution. Although average wage and industrial structure have been controlled, other variables related to urban development may still be omitted. These variables are positively correlated with air pollution and labor migration, making the negative impact of air pollution underestimated. In order to solve the endogeneity problem, the ventilation coefficient is used as an instrumental variable of air pollution, and the control function method is applied in a nonlinear model with continuous endogenous explanatory variables (Table 3).

The results of OLS regression in step l show that the coefficient of the instrumental variable (In(ve)) is negative at the 1% significance level, indicating that a more significant ventilation coefficient lowers the concentration of air pollution. It is in line with the expectation. However, it does not mean there is no weak instrument presence. One method to examine the weak instrument problem is to test whether the coefficient of IV in the first stage regression is 0. If the F value of the test is greater than 10, then the null hypothesis that the coefficient of the IV is 0 can be rejected. The first stage regression shows that the F-value (44.25) is greater than 10, indicating that the IV or ventilation coefficient is not weak.

In addition, the OLS regression of the ventilation coefficient on other city-specific variables in Appendix A shows that the coefficients of city-specific variables are not statistically significant except for the average wage of employees. The coefficient of the average wage is only statistically significant but has no practical significance. The results in Appendix A and the characteristics of the ventilation coefficient indicate that the instrumental variable is likely excludable. In general, the choice of the instrumental variable is reasonable.

The results of conditional logit regression in step 2 show that for every 10 μg/m^3^ increase in a city’s PM_2.5_ concentration, the odds of migrants choosing that city will be significantly reduced by 21.2%, which is higher than the original 9.7%. It implies that the direction of omitted variable bias is accurately predicted. The model (3) in Table 3 underestimates the negative impact of air pollution.

### 4.2. Robustness Tests

The questionnaire of CLDS also asked about the reasons for migration, including joining the army, supporting remote places, going down to the countryside, demolition and relocation, moving with family members, entrepreneurship, job search, and further studying. According to this question, this paper draws 3389 subsamples from the dataset, and their migration purposes are job or education. These individuals migrate to 240 cities in China; that is, they choose migration destinations among 240 prefecture-level cities in China. Why do we choose these two reasons? Because other reasons, such as supporting remote places and moving together with family, are easily influenced by government policy or other family members, their migration decision may be passive, not active. Furthermore, if people want to find a better job or education for themselves through migration, they will think more about the benefits and risks of migration.

Table 4 shows the result of subsample regression. It indicates that by holding the values for other alternatives constant, increasing the air pollution concentration by 10 μg/m^3^ for a given city decreases the odds of choosing that city by 24.3%. It implies that the negative impact of air pollution is robust. In addition, the conditional model with instrumental variables has a larger air pollution coefficient, which means the negative effect of air pollution is underestimated. 

The previous studies show that the distance between the origin and destination will affect the migration cost. On the one hand, the longer the distance, the higher the transportation cost. On the other hand, staying away from relatives and friends also decreases happiness. Therefore, if other things are equal, people may choose the destinations closer to their current regions of residence. Therefore, this paper further considers the influence of migration origin. A dummy variable indicating whether the city of origin and the alternative city are in the same province is included. Table 5 reports the results from conditional logit regression without and with IV, respectively. It indicates that migrants tend to choose destinations in the same province of their origin. After considering the origin’s impact, the coefficient of air pollution remains significantly negative.

### 4.3. Heterogeneity Analysis

In the previous analysis, all migrants are regarded as individuals with the same preference for air pollution. In this part, we will consider the individual heterogeneity, that is, the impact of age, gender, marriage, education, household registration, and family level on air pollution and migrants. Since the variables of personal characteristics cannot be directly added in the conditional logit model, this paper divides the samples into groups according to personal characteristics. For simplicity, this paper directly reports the odds ratios of air pollution in the following part and no longer reports the raw coefficients of all variables.

First, this paper will examine whether migrants with different ages, gender, and marital status have different sensitivity to air pollution. The samples are divided into 3 age groups, including 15–29 years old, 30–44 years old, and 45–64 years old. The results in Table 6 show that people aged between 30 and 44 are the most sensitive to air pollution. If a city’s PM_2.5_ concentration increases by 10 μg/m^3^, the odds of migrants choosing that city will decrease by 25.9%. The reason may be that the younger groups pay more attention to economic factors such as wages and employment opportunities, while the older groups receive less information and know little about the side effect of air pollution. The work of middle-aged groups has been relatively stable, and they have begun to care more about life quality. Therefore, they are the most sensitive to air pollution.

Regarding gender, the impact of air pollution on male migrants is higher than on female migrants. For male migrants, increasing the air pollution concentration by10 μg/m^3^ for a given city decreases the odds of choosing that city by 24.5%, which is greater than the 18.8% for females. The possible reason is that male migrants face fewer constraints in the labor market than female migrants, so they can think more about air pollution.

As for marital status, migrants with spouses are more sensitive to air pollution. Specifically, for every 10 μg/m^3^ increase in PM_2.5_ concentration in a city, the odds of migrants with spouses choosing that city significantly decrease by 22.1%. However, the odds of migrants without spouses only decrease by 12.7%, which is significant at the 10% level. It shows that, compared with migrants without spouses, migrants with spouses not only consider themselves but also consider the health of their family members, so they will be more concerned about the negative effects of air pollution.

Second, this paper will study the impact of air pollution on migrants by education level, household registration, and family level. The grouped regression by education level shows that the impact of air pollution on migrants with a junior college education and above is more significant than on those with high school and below. It may be because higher education people have a more comprehensive understanding of the negative effect of air pollution and thus become more sensitive to it. Furthermore, these people have more or better employment opportunities and are less affected by the labor market, so they care more about life quality.

Considering the influence of household registration on migrants, we divided it into two groups according to whether the migration destination and the location of household registration are in the same province or not. It is found that individuals whose migration destination is in the different province of household registration are more sensitive to air pollution. It may be because these people consider more about the restrictions due to household registration, and the requirements for air quality are relatively low. 

Family factors also affect migrants’ sensitivity to air pollution. We use the following item in the questionnaire to measure the family level: What level do you think your family was when you were 14 years old? We further divide the family level into two groups: more than 5 score and less than 5 score. The results in Table 7 show that for migrants with a high self-rated family level, increasing a city’s PM_2.5_ concentration by 10 μg/m^3^ lowers the odds of migrating to that city by 23.4%. The negative impact of air pollution on the low self-assessment level of migrants’ households is even smaller (18.5%). Migrants with a low self-rated family level may pay more attention to economic factors such as employment and wage, so they are less affected by air pollution.

In general, the results of grouped regression based on individual characteristics show that the negative impact of air pollution on migrant migration is still statistically significant, indicating that the results are robust. At the same time, there is individual heterogeneity in the impact of air pollution on migrants. Middle-aged, male, married, or highly educated groups are more sensitive to air pollution when choosing migration destinations. Household origin and family level will affect the sensitivity, as well. Finally, air pollution affects the movement of migrants, which will change the social demographic composition of a city.

## 5. Discussion

With the improvement in living conditions, people have begun to pay more attention to air quality and know more about the side effects of air pollution. However, there exists a contradiction between the requirements for better air quality and the current status of severe air pollution. Residents will take lots of avoidance behavior to avoid the adverse effects of air pollution. As for migrants, if other things are equal, they may choose to move to cities with better air quality to reduce their exposure to air pollution. Therefore, this paper decides to study the impact of air pollution on migrants’ choice of destination city. Assessing the impact of air pollution on migrant migration is significant for local governments to design policies to attract migration inflows.

In the empirical analysis, this paper matches the China Labor Force Dynamic Survey data with the air pollution data of 267 prefecture-level cities. Then, a dataset about migration choices among 267 cities in China is constructed. Since the dependent variable is qualitative and has 267 options, this paper uses the conditional logit model to analyze the regression. Considering that air pollution is endogenous and omitted variables may bias estimates, this paper uses the ventilation coefficient as an instrument for air pollution. Moreover, the conditional logit model is nonlinear, and 2SLS is no longer appropriate, so the control function method is applied to deal with the problem. 

This paper have essential policy significance for local governments. From the conclusions of this paper, severe air pollution reduces migration probability, and highly educated people are more sensitive to air pollution. Therefore, governments can attract talent by improving the quality of the environment and, thus, accumulate more human capital. Unlike the stage of high growth, the high-quality development of the economy depends heavily on talent. Apart from wages and benefits, air quality also plays an essential role in attracting talented individuals. Meanwhile, an improved environment is more conducive to inter-regional migration. Therefore, the government can improve the quality of the environment to encourage more migrants to migrate across regions and inject new vitality and development into local areas. To promote high-quality development, local governments should integrate environmental policy, human resource management, and economic growth. In addition, the conclusions of this paper also provide ideas for how to promote the balanced distribution of labor. As mentioned, the migrant population tends to gather in large cities and causes many inconveniences. Even though wages and employment opportunities are still the most important factors that attract migration inflows, people are also paying more attention to public services and environmental quality. Therefore, improving the quality of the environment and promoting the equalization of public services, to some extent, can reduce the excessive gathering of populations in large cities. Then, the management pressure of large cities is reduced, and regional coordinated development is improved.

This paper provides new empirical evidence for air pollution and migrant migration research. First, the dependent variable of this paper is the destination choice of migrants, rather than the number of migrants in each province and city. That is chosen in order to study the impact of air pollution on labor migration at the micro level. Second, the paper no longer overly focuses on the economic or political factors but pays more attention to the city’s livability, which aligns with future development trends. Finally, the ventilation coefficient is used as an instrument for air pollution to identify the pure effect of air pollution. Meanwhile, the control function method is used to solve the problem that 2SLS cannot be applied to nonlinear models. However, this paper still needs to make further efforts in the following aspects: First, if there is a micro-database that tracks the movement of the migrant population, the method of panel data can be used for further research. It can also explore whether the city’s selection by the same migrant changes and what the reasons are for the changes. If other countries or regions have similar databases, their situation can be compared with China. Secondly, other air pollution proxies and instrumental variables should be tried, such as considering wind direction to build a new instrumental variable. As mentioned above, China’s air pollution data may be inconsistent with the actual situation. If there are other reliable air pollution data, different air pollution proxies can be used for research and comparison. Additionally, researchers can try to find better instruments for air pollution or exploit some policy changes to assess the causal effect. Different cities may have different environmental policies, so these cities are naturally divided into two groups. Then, a quasi-experimental method to identify the impact of air pollution should be used.

## 6. Conclusions

Generally, ceteris paribus, the more serious the air pollution in a city, the lower the probability of migrants choosing to flow into the city. Specifically, after considering the endogeneity of air pollution and controlling other city-specific variables, this paper finds that if the PM2.5 concentration of a city increases by 10 μg/m^3^, the odds of migrants choosing to move to the city will decrease by 21.2%. Moreover, the results are robust to different specifications, including using different samples and adding the impact of migration origin. Therefore, when the number of flowing populations enters the adjustment period, good environmental quality can also become essential in attracting and retaining talents. At last, this paper divides the samples into different groups according to age, gender, marital status, education level, location of household registration, and self-rated family level. It is found that different migrants have different sensitivity to air pollution. Male/middle-aged/married/highly educated people are more sensitive to air pollution. Household origin and the family level also affect people’s sensitivity to air pollution. The individual heterogeneity of air pollution may lead to changes in the sociodemographic composition of the labor force of China’s cities.

## Figures and Tables

**Table 1:** Descriptive statistics of variables.

Variables	Obs	Mean	Std	Min	Max
Age	6115	37.67	11.29	15	64
Gender (male = 1)	6115	0.47	0.49	0	1
Marriage (married = 1)	6115	0.87	0.34	0	1
Education year	6115	9.43	3.98	0	22
Family level	6115	3.12	1.87	0	10
*PM*_2.5_ concentration (10 μg/m^3^)	267	3.31	1.61	0.30	7.47
Average temperature (°C)	267	13.98	5.11	−1.60	25.82
Precipitation (100 mm)	267	8.32	4.79	0.14	21.10
Ln(ve) (Ln(ve) represents the log of Ventilation Coefficient) (IV)	267	6.43	0.43	5.52	8.04
Average wage (1000 yuan)	267	4.98	1.30	3.21	10.80
Industrial structure (non-agricultural proportion, %)	267	83.71	8.98	56.83	98.84
Educational level (teachers per 1000 people)	267	8.23	1.68	5.12	20.24
Medical level (hospital beds per 1000 people)	267	3.11	1.32	1.27	9.33
Ln (population) (Ln (population) represents the log of population size)	267	7.92	0.81	5.21	11.11
Whether the city is provincial capital	267	0.11	0.32	0	1
Whether the origin city and the alternative city is in the same province	606,891	0.05	0.21	0	1

Notes: The variable of whether the origin city and the alternative city are in the same province varies with different individuals, so the observations are calculated by sample size times the number of alternative cities, namely (2273 × 267) observations. Since only the data from 2012 has the information of origin, the sample size is 2273.

**Table 2:** Conditional logit regression without using IV.

	Model 1		Model 2		Model 3	
	Raw Coefficient	% Change in Odds	Raw Coefficient	% Change in Odds	Raw Coefficient	% Change in Odds
*PM*_2.5_	0.059 *** (0.008)	6.1	−0.042 *** (0.007)	−4.0	−0.102 *** (0.011)	−9.7
Temperature	0.069 *** (0.004)	7.1	0.091 *** (0.005)	9.6	0.067 *** (0.007)	6.9
Precipitation	0.055 *** (0.005)	5.6	0.021 *** (0.006)	2.1	0.035 *** (0.006)	3.6
Average wage			0.237 *** (0.009)	26.9	0.186 *** (0.013)	20.4
Industrial structure			0.068 *** (0.004)	7.1	0.038 *** (0.003)	3.9
Educational level					0.055 *** (0.007)	5.6
Medical level					0.225 *** (0.014)	25.2
Population size					0.434 *** (0.025)	54.5
Provincial capital					0.493 *** (0.038)	63.8
Chi^2^	1279.65		4779.63		9089.78	
R^2^	0.031		0.095		0.201	
*N*	1,632,705		1,632,705		1,632,705	

Notes: Robust standard errors are in brackets and *** *p* < 0.01 (the same below).

**Table 3:** Conditional logit regression by using Ⅳ.

Step 2: Clogit		
	Raw Coefficient	% Change in Odds
PM_2.5_	−0.238 *** (0.021)	−21.2
Residual from step 1	0.187 *** (0.027)	20.5
Temperature	0.077 *** (0.005)	8.2
Precipitation	0.026 *** (0.007)	2.7
Average wage	0.178 *** (0.011)	19.5
Industrial structure	0.046 *** (0.003)	4.7
Educational level	0.030 *** (0.008)	3.1
Medical level	0.241 *** (0.012)	27.3
Population size	0.491 *** (0.032)	63.9
Provincial capital	0.374 *** (0.044)	45.3
Chi2	9679.47	
R^2^	0.115	
Step 1: OLS regression of air pollution on ventilation coefficient		
Ln(ve)	−1.140 *** (0.171)	
Control variables	Yes	
F-value	44.25	
R^2^	0.491	
*N*	1,632,705	

Note: *** *p* < 0.01.

**Table 4:** Robustness check l: reasons for migration.

Clogit + IV		
	Raw Coefficient	% Change in Odds
PM_2.5_	−0.279 *** (0.029)	−24.3
Residual from step 1	0.211 *** (0.039)	23.5
Temperature	0.063 *** (0.009)	6.5
Precipitation	0.067 *** (0.008)	6.9
Average wage	0.168 *** (0.013)	18.3
Industrial structure	0.071 *** (0.005)	7.3
Educational level	0.039 *** (0.011)	4.0
Medical level	0.253 *** (0.021)	28.9
Population size	0.547 *** (0.049)	72.8
Provincial capital	0.251 *** (0.061)	28.5
Chi^2^	7478.76	
R^2^	0.172	
Clogit		
*PM*_2.5_	−0.142 *** (0.017)	−13.2
Control variables	Yes	
Chi^2^	7234.67	
R^2^	0.171	
*N*	813,360	

Note: *** *p* < 0.01.

**Table 5:** Robustness check 2: the impact of origin.

	(1) Clogit		(2) Clogit + IV	
	Raw Coefficient	% Change in Odds	Raw Coefficient	% Change in Odds
PM_2.5_	−0.151 *** (0.020)	−14.1	−0.401 *** (0.045)	−33.0
Whether in the same province or not	1.143 *** (0.041)	213.6	1.232 *** (0.048)	242.8
Residual from step 1			0.301 *** (0.043)	35.1
Control variables	Yes		Yes	
Chi^2^	6798.64		7212.33	
R^2^	0.271		0.283	
*N*	606,891		606,891	

Note: *** *p* < 0.01.

**Table 6:** Individual heterogeneity: age, gender, marriage.

	Age			Gender		Marital Status	
	15–29	30–44	45–64	Male	Female	Married	Unmarried
Clogit + IV							
PM_2.5_	−0.246 *** (0.041)	−0.259 *** (0.027)	−0.201 *** (0.034)	−0.245 *** (0.027)	−0.188 *** (0.021)	−0.221 *** (0.023)	−0.127 * (0.062)
Control variables	Yes	Yes	Yes	Yes	Yes	Yes	Yes
Chi^2^	3479.42	4085.22	2352.78	5161.62	4597.24	4689.20	1263.42
R^2^	0.161	0.128	0.077	0.125	0.106	0.104	0.168
Clogit							
PM_2.5_	−0.092 *** (0.021)	−0.138 *** (0.016)	−0.066 *** (0.016)	−0.120 *** (0.014)	−0.076 *** (0.013)	−0.127 *** (0.014)	−0.115 *** (0.033)
Control variables	Yes	Yes	Yes	Yes	Yes	Yes	Yes
Chi^2^	3398.08	3811.42	2128.63	4871.08	4292.64	4433.46	1248.21
R^2^	0.158	0.125	0.076	0.124	0.103	0.102	0.164
*N*	432,006	642,669	558,030	804,738	827,967	1,374,249	258,456

Note: *** *p* < 0.01, * *p* < 0.1.

**Table 7:** Individual heterogeneity: education level, household origin, and family level.

	Education Level		Household Origin		Family Level	
	High School and Below	Junior College and Above	Same Province	Different Provinces	≥5	<5
Clogit + IV						
PM_2.5_	−0.184 *** (0.039)	−0.244 *** (0.021)	−0.146 *** (0.031)	−0.328 *** (0.041)	−0.234 *** (0.021)	−0.185 *** (0.033)
Control variables	Yes	Yes	Yes	Yes	Yes	Yes
Chi^2^	7212.63	2449.54	2821.54	2638.30	3347.78	6321.56
R^2^	0.109	0.152	0.073	0.312	0.128	0.108
Clogit						
PM_2.5_	−0.089 *** (0.009)	−0.118 *** (0.019)	−0.166 *** (0.014)	−0.211 *** (0.032)	−0.095 *** (0.012)	−0.097 *** (0.014)
Control variables	Yes	Yes	Yes	Yes	Yes	Yes
Chi^2^	7104.09	2343.55	2627.53	2628.10	3176.38	5968.22
R^2^	0.106	0.151	0.073	0.308	0.129	0.107
*N*	1,282,935	349,770	801,000	64,320	539,874	1,092,831

Note: *** *p* < 0.01.

## Data Availability

Restrictions apply to the availability of these data because the data were obtained from a third party. It could be available from the corresponding authors (Q.W. and J.Z.) with the permission of the third party.

## Appendix A. An Auxiliary Check on the Excludability of VC

**Table A1:** Determinants of VC.

Variables	Dependent Variable: ln(vc)
Temperature	0.002 (0.008)
Precipitation	−0.005 (0.011)
Average wage	−0.212 *** (0.053)
Industrial structure	−0.003 (0.006)
Educational level	0.021 (0.017)
Medical level	−0.007 (0.034)
Population size	−0.118 (0.038)
Provincial capital	−0.020 (0.119)
*N*	267
R^2^	0.252

Note: *** *p* < 0.01.
